# Cattaneo–Christov heat flow model for copper–water nanofluid heat transfer under Marangoni convection and slip conditions

**DOI:** 10.1038/s41598-022-09275-w

**Published:** 2022-03-30

**Authors:** Khalid Abdulkhaliq M. Alharbi, Mohammed Nasser Alshahrani, Naeem Ullah, Naseer M. Khan, Krawczuk Marek, Abd Allah A. Mousa, Sajid Ali

**Affiliations:** 1 Mechanical Engineering Department, College of Engineering, Umm Al-Qura University, Makkah, Kingdom of Saudi Arabia; 2 Department of Mathematics, College of Science and Humanities in Al-Kharj, Prince Sattam bin Abdulaziz University, Al-Kharj, 11942 Saudi Arabia; 3 Department of Mathematics, Quaid-i-Azam University, 45320 Islamabad, Pakistan; 4 School of Mathematics and Statistics, Central South University, Changsha, 410083 Hunan People’s Republic of China; 5 Gdansk University of Technology, Faculty of Mechanical Engineering and Ship Technology, Narutowicza 11/12, 80-233 Gdańsk, Poland; 6 Department of Mathematics and Statistics, College of Science, Taif University, P.O. Box 11099, Taif, 21944 Saudi Arabia; 7 Mechanical and Energy Engineering Department, College of Engineering, Imam Abdulrahman Bin Faisal University (IAU), Dammam, Kingdom of Saudi Arabia

**Keywords:** Engineering, Materials science, Mathematics and computing

## Abstract

This report is devoted to the study of the flow of MHD nanofluids through a vertical porous plate with a temperature-dependent surface tension using the Cattaneo–Christov heat flow model. The energy equation was formulated using the Cattaneo–Christov heat flux model instead of Fourier’s law of heat conduction. The Tiwari–Das model was used to take into account the concentration of nanoparticles when constructing the momentum equation. The problem is described mathematically using the boundary layer approach as a PDE, which is then converted into an ODE with the help of the transformation process. The solution finding process was completed by running the bvp4c code in MATLAB. A quantitative analysis of the influence of some newly occurring parameters on physical quantities was carried out using graphics. The addition of nanoparticles to the base fluid leads to an increase in both skin friction and thermal conductivity. The increase in thermal conductivity is the advantage, while the increase in skin friction is the disadvantage of the nanoparticle concentration. Marangoni convection has proven to be one of the most cost-effective tools available that can reduce skin friction. Marangoni convection improves the heat transfer coefficient during suction but decreases the heat transfer coefficient during the injection.

## Introduction

The traditional Fourier law of heat transfer^1^ is the most reliable approach to understanding the dynamics of heat transfer under various conditions. Nevertheless, it has a fundamental flaw: it requires the establishment of a parabolic equation for the energy of the temperature field, which is not compatible with the principle of causality. In its well-known study, Cattaneo^2^ offered a successful adaptation of the Fourier model in order to add an essential feature of the thermal relaxation time. It can be seen from this that a hyperbolic energy equation is formed for a temperature field, which makes it possible to transfer heat at a limited speed via the propagation of heat waves. This type of heat transfer has interesting practical applications ranging from nanofluidic flows to modeling skin burns (it is desirable to see^3^). It has been found that thermal relaxation times for some materials, including biological tissue (1–100 s), sand (21 s), and $\text{NaHCO}_3$ (29 s), are long. To preserve the material-invariant calculation, Christov^4^ replaced the time derivative with the upper-convected derivative of Oldroyd in the Maxwell-Cattaneo’s model. This model is referred to in the literature as Cattaneo–Christov heat flow model. Garia^5,6^ studied in detail the steady MHD flow of a hybrid nanofluid, considering multiple geometries such as wedge and cone. The natural convective boundary layer flow in the Newtonian liquid was investigated by Straughan^7^. The heat transfer study of a Maxwell fluid under the velocity slip condition according to the Cattaneo–Christov approach is carried out by Han^8^. They used methods of Homotopy analysis and a numerical method, the so-called finite difference scheme, to find solutions to the basic equations.

The combined effect of magnetic field and biological convection on the boundary layered flow of unsteady MHD Sakiadis and Blasius nanofluids was investigated by Ali^9^. Ibrahim^10^ published a communication report on viscoelastic nanofluid flow related to the Cattaneo–Christov mass and heat flow model and third-order velocity slip, and investigated various relevant flow parameters. Using a new mathematical model of a second-grade bioconvective nanofluid in combination with viscous dissipation and the Cattaneo–Christov heat flow model for the transfer of heat through a permeable medium, Raja^11^ initiated a study with the Levenberg-Marquardt technique (LMT) on the smooth backpropagated Neural Networks (BNN) calculation. The effect of MHD on the rotational flow of the unsteady Oldroyd-B nanofluid with the concentration and temperature of the nanoparticles is related to Brownian motion and the Cattaneo–Christov heat flow is explained by Ali^12^. With the Cattaneo–Christov heat flow, the VON Kármán spinning flow problem is extended to the slip condition of Navier on the stretched spinning disk surface. Lim^13^ examined semi-analytically the flow of an MHD Casson liquid over a rotating disk. Farooq’s research is aimed at obtaining numerical results for an MHD nanofluid flow with a Cattaneo–Christov heat flow model and thermal radiation through a stretched surface with melting boundary conditions. The Cattaneo–Christov model is used for a 2D boundary layer. Salahuddin’s study^14^ uses the nanofluid cross-flow across the surface of a parabola and the temperature-dependent viscosity to investigate heat and mass diffusion. In addition, the study of the influence of changes in enthalpy and activation energy broadens the scope of this topic. Mushtaq^15^ considered the Cattaneo model, which is an improved form of the Fourier equation for heat conduction, including thermal inertia.

When surface tension is high due to temperature and material gradients, Marangoni convection occurs. Microgravity is dominated by Marangoni convection and its applications include heat exchangers, crystal growth, coating processes, soap film stabilization, silicon wafer growth and other technical applications^16^. Using a series of three-dimensional numerical calculations that take into account the effect of radiative heat transfer to the free surface, the properties of Marangoni convection in a shallow rectangular cavity subject to mutually perpendicular gradients of temperature and concentration are investigated^17–21^. Wang^22^ experimentally investigated the tendencies of Marangoni convection in an evaporating droplet deposited over a volatile liquid layer. At two different equilibrium states, two types of Marangoni instabilities are found on the surface of a methanol droplet. In the entire floating zone with radiant heat transfer under zero gravity conditions, Chihao Jin^23^ carried out a computational study of the Marangoni thermosolar convection. Sun^24^ has developed an axisymmetric 2D mathematical model to predict the melting of spherical EPCMs, including air pockets at the top. In addition to natural convection due to buoyancy, this topic also discusses for the first time Marangoni convection due to thermocapillary force at the PCM interface with molten air. The entropy nature of the laminar, steady, relative contribution of solitary and thermal Marangoni convection during the passage of a hybrid Cassonian nanofluid $(\text{Al}_2\text{O}_3\text{-Cu-H}_2\text{O})$ in a disk flow under the action of a nonlinear heat source/sink, radiation, viscous dissipation and nonlinear convection are described in the Yun-Xiang Li’s^25^ study.

Microfluidics, semiconductor vapor drying in microelectronics, foams, surfactant replacement therapy for newborns, coating technology and film drainage in emulsions are examples of Marangoni convection, which is important in industrial, biomedical and everyday applications. Song’s^26^ research focuses on the role of bioconvection in the flow of Carreau nanofluid through a stretched cylinder. The study was modified to include the effects of melting and chemical reactions. Zhang^27^ used a series of 3D numerical models to study the features of Marangoni convection in a narrow rectangular channel with a linear boundary condition. In his work, Mackolil^28–33^ explains many aspects of Marangoni convection on the macroscopic properties of nanofluids by considering different geometries with new boundary conditions and fluid models. Kazemi^34^ developed a study to investigate the formation of entropy in hybrid nanofluids, including parts of Marangoni convection and a Darcy-Forchheimer model to explain the momentum equation.

In the current study, the authors examined the flow of a viscous nanofluid on a vertical porous plate with the effects of the magnetic field, suction, and Cattaneo–Christov heat flux. The effect of Marangoni convection and volume fraction during heat transfer was investigated using the Tiwari–Das model and the Cattaneo–Christov model. Many scientists, including Farooq^35^, have worked on a comparative problem of fluid flow through a vertical porous plate, but none have related Marangoni convection and Cattaneo–Christov heat flow in their research. When the Cattaneo–Christov heat flow and slip conditions are included, fluid problems are appropriately addressed in the real scenario. The assumption that a liquid flows through a porous plate is critical to many industrial and technological activities, including power generation, turbomachinery, and food processing. The stated purpose of the analysis is:

- The equations of concentration and energy are modified using the connections of the Cattaneo–Christov theory.
- The purpose of this study is to see how Marangoni convection affects the thermal transport of nanofluid.
- To see how velocity slip affects the thermal transport of nanofluid.
- The effect of the Prandtl number on the effective Prandtl method, as well as the suction phenomenon, was investigated.
- The concentration, velocity and temperature of the nanofluid are analyzed using 2D graphical analysis.

## Mathematical modeling

**Figure 1:**
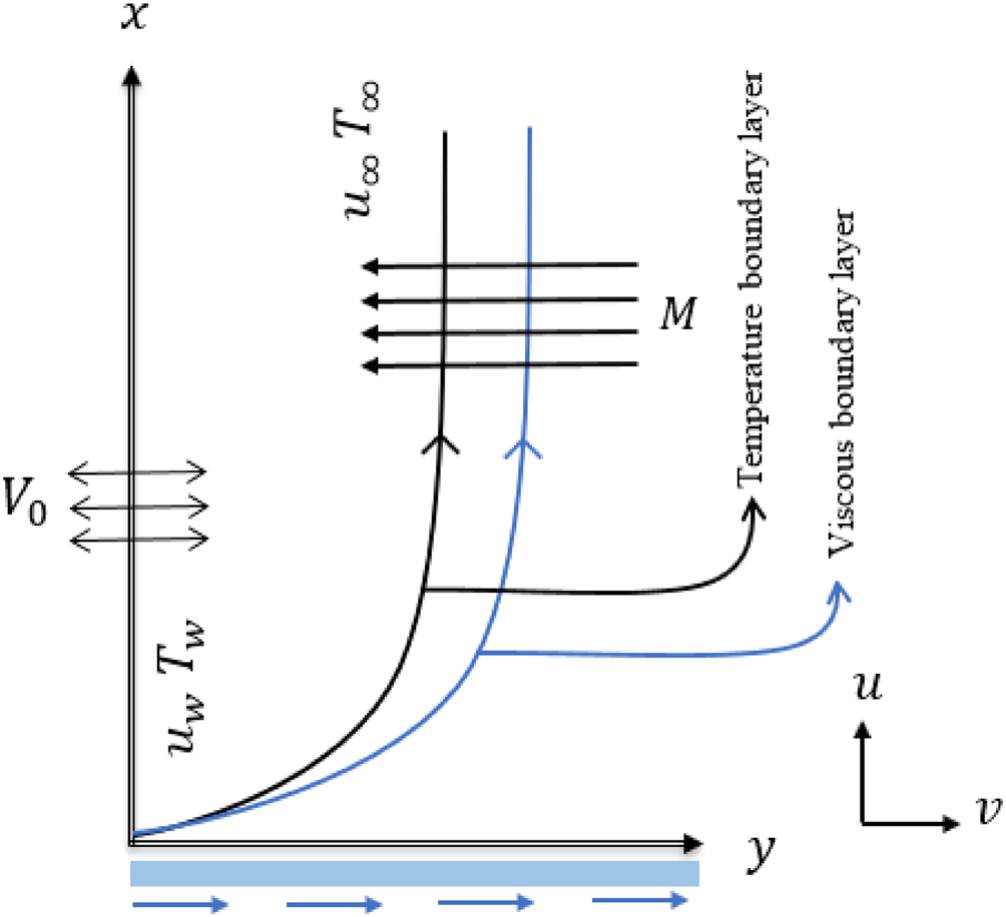
Flow model exhibition.

In the Cartesian plane, it is proposed to study the Marangoni convection of Copper–Water MHD nanofluids with temperature-dependent surface tension and Cattaneo–Christov heat flux. The flow at the surface of a porous vertical plate is considered under the assumption that there is a constant suction $v_0$ at the surface. Suction is a boundary layer control approach aimed at reducing energy losses in channels or drag on bodies in an external flow. The nanofluid investigated was prepared by immersing copper nanoparticles in water and was called the Tiwari–Das model^36^ for constructing the equation of momentum. The nanofluid flow is magnetized by passing a constant magnetic flux perpendicular to the nanofluid flow (see Fig. 1). Due to the low Reynolds number, the strength of the induced magnetic field is negligible and the induced magnetic field does not affect heat transfer. The current study assumes that the viscosity and density of the fluid are independent of temperature. The velocity of nanofluid is zero if the distance from the sheet is significant and the temperature at this point is considered equal to $T_{\infty }$, and the surface temperature of the sheet is expressed as $T_{w}$. The momentum and temperature equations that govern the current flow model can be approximated by boundary layer approximation as follows^37,38^:

1 $$\begin{aligned} \frac{\partial u}{\partial x} + \frac{\partial v}{\partial y} =&0, \end{aligned}$$

2 $$\begin{aligned} u \frac{\partial u}{\partial x} + v \frac{\partial u}{\partial y} =&\left( \frac{\mu }{\rho }\right) _{nf} \frac{\partial ^2 u}{\partial y^2} - \left( \frac{\sigma }{\rho }\right) _{nf} {B_0}^2 u, \end{aligned}$$

3 $$\begin{aligned} u \frac{\partial T}{\partial x} + v \frac{\partial T}{\partial y} =\,&\alpha _{nf} \frac{\partial ^2 T}{\partial y^2}- \lambda _T \left( u^2 \frac{\partial ^2 T}{\partial x^2} + v^2 \frac{\partial ^2 T}{\partial y^2} + 2uv \frac{\partial ^2 T}{\partial x \partial y} + u \frac{\partial u}{\partial x} \frac{\partial T}{\partial x} +\right. \nonumber \\&\left. u \frac{\partial v}{\partial x} \frac{\partial T}{\partial y} + v \frac{\partial u}{\partial y} \frac{\partial T}{\partial x} + v \frac{\partial v}{\partial y} \frac{\partial T}{\partial y}\right) , \end{aligned}$$

The Cartesian velocity components in the $x$- and $y$- directions are indicated as *u* and *v*, respectively. Thermal diffusivity of nanofluid is represented as $\alpha _{nf}$, density is represented as $\rho _{nf}$, and dynamic viscosity is represented as $\mu _{nf}$. The indices f and nf indicate the base fluid and nanofluid. The thermal relaxation time of the fluid is specified by $\lambda _T$. The main thermophysical properties of the nanofluids investigated are taken from the standard literature. The combination of equations that can estimate the physical aspects of the flow model is related to the following boundary conditions:

4 $$\begin{aligned} \left. \begin{array}{ll} \mu _{nf} \frac{\partial u}{\partial y} = - N \frac{\partial \sigma _m}{\partial x} = - N \frac{\partial \sigma _m}{\partial T} \frac{\partial T}{\partial x}, v=v_0, T = T_w = T_\infty + T_0 \left( \frac{x}{l}\right) ^2 \; \text {at} \; y = 0, \\[5pt] u \longrightarrow 0, v \longrightarrow 0, T \longrightarrow T_\infty \; \text {as} \; y \longrightarrow \infty . \end{array} \right\} \end{aligned}$$

The coefficient of surface tension, which is a function of temperature, can be formulated by the following equation^39^:

5 $$\begin{aligned} \left. \begin{array}{ll} \sigma _m = \sigma _0 \left[ 1 - \gamma _T (T - T_\infty )\right] , \\ \gamma _T = \frac{-1}{\sigma _0}\frac{\partial \sigma _m}{\partial T}|_{T=T_\infty }. \end{array} \right\} \end{aligned}$$

The parameters occurring in the boundary conditions can be explained by the fact that *N* is the slip coefficient, $\sigma _m$ is the surface tension and *l* is the characteristic length. The following are the Similarity Transformations used in this article to convert the partial differential equations to regular ordinary ones^37^.

6 $$\begin{aligned} \left. \begin{array}{ll} u = ax f^{\prime }(\eta ), v = \left( a \nu _f\right) ^{\frac{1}{2}} f(\eta ), \\ T = T_\infty +(T_w - T_\infty )\theta (\eta ), \eta = \left( \frac{a}{\nu _f}\right) ^{\frac{1}{2}} y. \end{array} \right\} \end{aligned}$$

The continuity equation is satisfied by applying the transformation defined above while converting the momentum and temperature equations into the following equations.

7 $$\begin{aligned}&\frac{\rho _f}{\rho _{nf} } \frac{\mu _{nf}}{\mu _{f}} f^{\prime \prime \prime } + f f^{\prime \prime } - (f^{\prime })^2 - \frac{\sigma _{nf}}{\sigma _f} \frac{\rho _f}{\rho _{nf}} M f^{\prime }=0, \end{aligned}$$

8 $$\begin{aligned}&\frac{k_{nf}}{k_f} \theta ^{\prime \prime } + \frac{(\rho c_p )_{nf}}{(\rho c_p )_f } Pr \left[ \left( f \theta ^{\prime } - 2 f^{\prime } \theta \right) + \lambda _1 \left( 5 f f^{\prime } \theta ^{\prime } + 2 f f^{\prime \prime } \theta - \theta (f^{\prime })^2 - f^2 \theta ^{\prime \prime }\right) \right] =0. \end{aligned}$$

Boundary conditions are transformed as follows:

9 $$\begin{aligned} \left. \begin{array}{ll} f (0) = S, f^{\prime \prime } (0) = -2 \lambda Ma (1- \phi )^{(-2.5)}, \theta (0) = 1, \\ f^{\prime } \longrightarrow 0 ,\theta \longrightarrow 0, \; \text {as} \; \eta \longrightarrow \infty . \end{array} \right\} \end{aligned}$$

The dimensionless parameters involved in Eqs. (7)–(9) are mathematically represented as:

10 $$\begin{aligned} \left. \begin{array}{ll} M = \frac{2 \sigma _f {B_0}^2}{\rho _f a}, Pr = \frac{(\mu c_p )_f}{k_f}, Ma = \frac{\gamma _T \sigma _0 T_0}{l^2 a \sqrt{a \rho _f \mu _{f}}}, \lambda _1 = a \lambda _T, \\ S = v_0 (c \nu )^{-\frac{1}{2}}, \lambda = a \sqrt{\frac{U_0}{N}} \end{array} \right\} \end{aligned}$$

## Numerical procedure

By including similarity variables, the corresponding partial differential equations and boundary conditions are first converted into a set of non-linear ordinary differential equations. The Eqs. (7), (8) and the boundary conditions (9) are then transformed into a system of ordinary differential equations of the first order, which are numerically solved in MATLAB using the bvp4c approach. The collocation approach has been used to solve the following problem. The procedure is explained as follows. Let $f(\eta ) = s_1, f^{\prime }(\eta ) = s_2, f^{\prime \prime }(\eta ) = s_3, \theta (\eta ) = s_4, \theta ^{\prime }(\eta )= s_5$. Then the set of equations written at the last of the mathematical modeling section as:

11 $$\begin{aligned} s s_1 (\eta ) =&\frac{\rho _{nf} \mu _{f}}{\rho _f \mu _{nf}} \left[ {(s_2)}^2 + \frac{\sigma _{nf}}{\sigma _f} \frac{\rho _f}{\rho _{nf}} M s_2 - s_1 s_3 \right] \end{aligned}$$

12 $$\begin{aligned} s s_2 (\eta ) =&\frac{- Pr (\rho c_p )_{nf}}{\left[ \frac{k_{nf}}{k_f} - \frac{{s_1}^2 Pr (\rho c_p )_{nf}}{(\rho c_p )_f } \right] (\rho c_p )_f} \left[ \left( s_1 s_5 - 2 s_2 s_4\right) + \lambda _1 \left( 5 s_1 s_2 s_5 + 2 s_1 s_3 s_4 \right. \right. \nonumber \\&\left. \left. - s_4 (s_2)^2 \right) \right] =0. \end{aligned}$$

13 $$\begin{aligned}&\left. \begin{array}{ll} s_1 (0) = S, s_3 (0) = -2 \lambda Ma (1- \phi )^{(-2.5)}, s_4 (0) = 1, \\[5pt] s_2 \longrightarrow 0 ,s_4 \longrightarrow 0, \; \text {as} \; \eta \longrightarrow \infty . \end{array} \right\} \end{aligned}$$

To find a solution, you need to run a residual test for accurate data transfer and grid-point selection when working with the bvp4c scheme in MATLAB. The convergence criterion for this particular numerical code is $1 \times 10^{-6}$. Dividing the difference between the edge points by the number of grid points gives the step size (i.e. $\frac{b-a}{n}$). If one of the boundary points is infinite, you must use the corresponding integer. In this case, the value $\eta = \eta _{\infty } = 10$ was used. Make good initial estimates to find the best solution to the Eqs. (11), (12) and related boundary condition problems.

## Discussion

**Figure 2:**
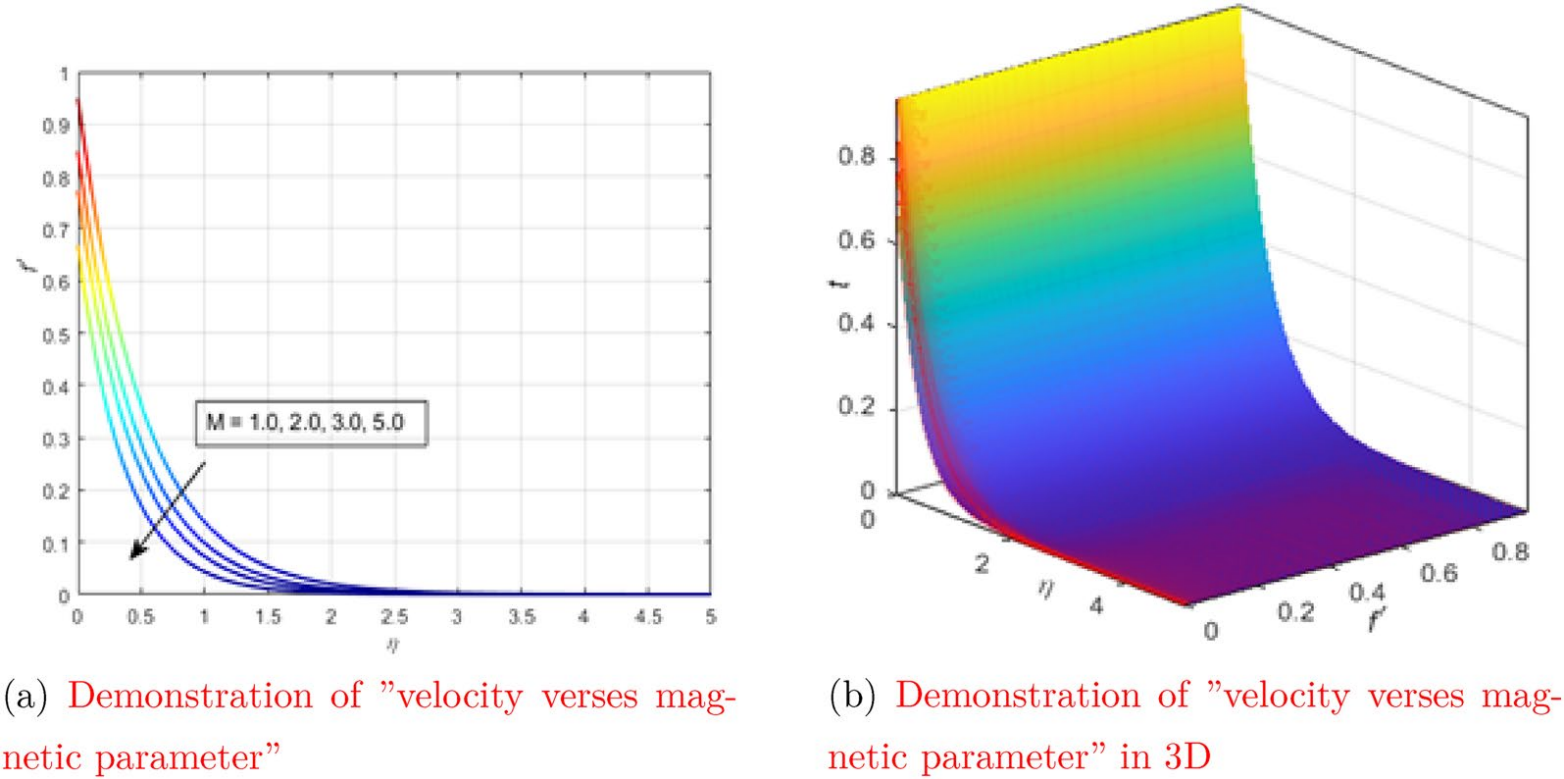
Demonstration of the dependence of velocity on the physical parameters in both 2D and 3D (see Appendix A).

**Figure 3:**
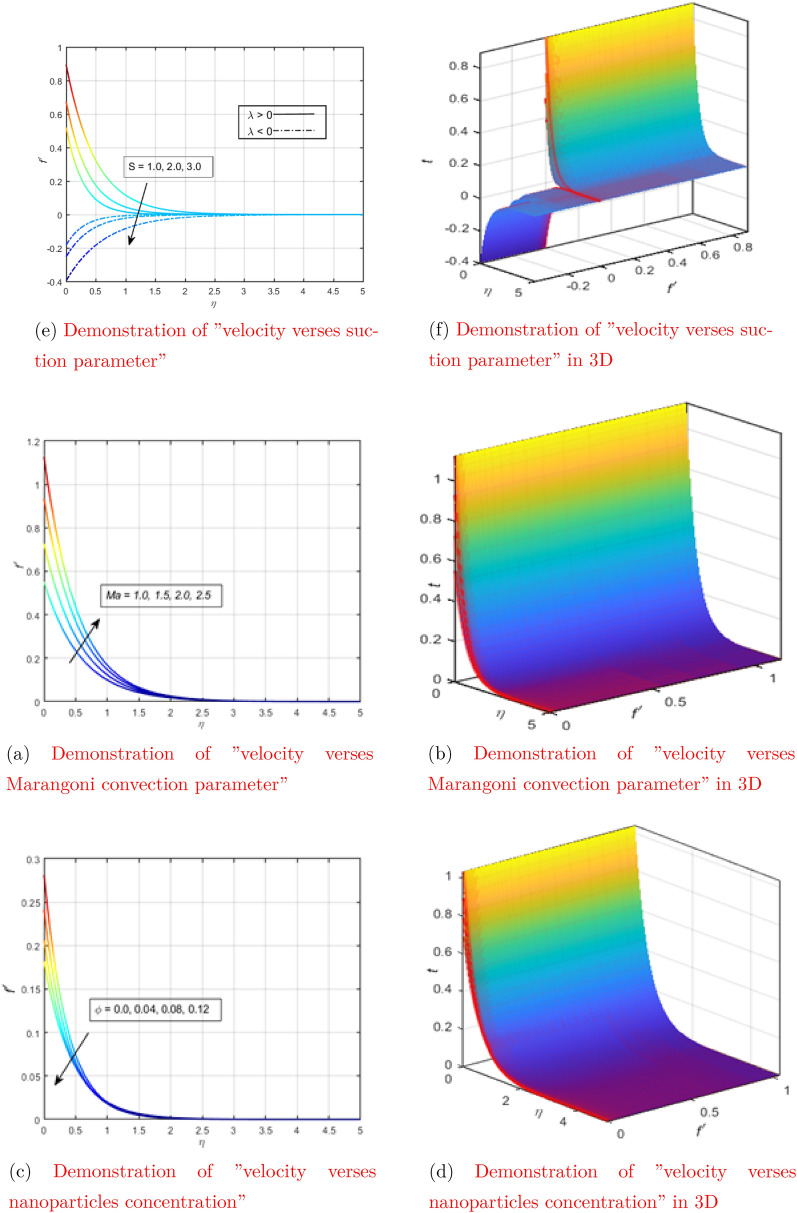
Demonstration of the dependence of velocity on the physical parameters in both 2D and 3D (see Appendix A).

**Figure 4:**
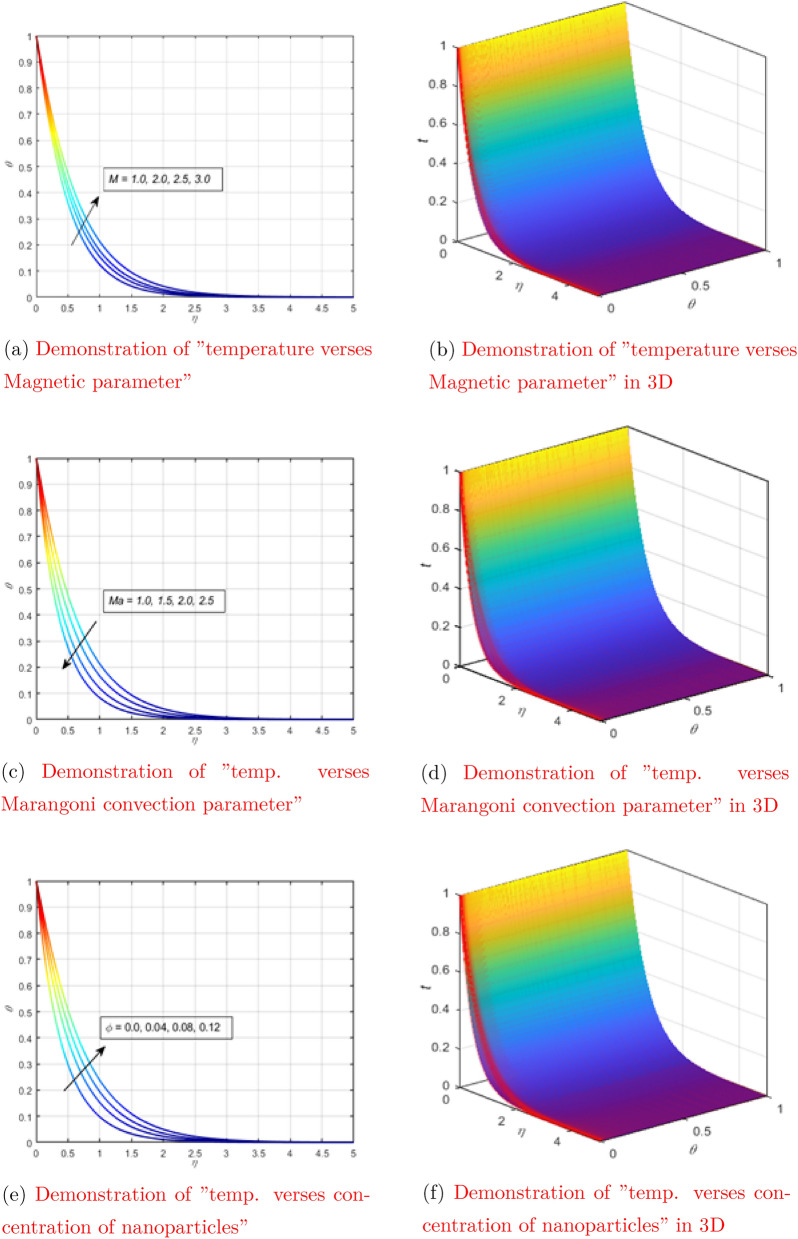
Demonstration of “temperature verses different physical parameters” (see Appendix A).

**Figure 5:**
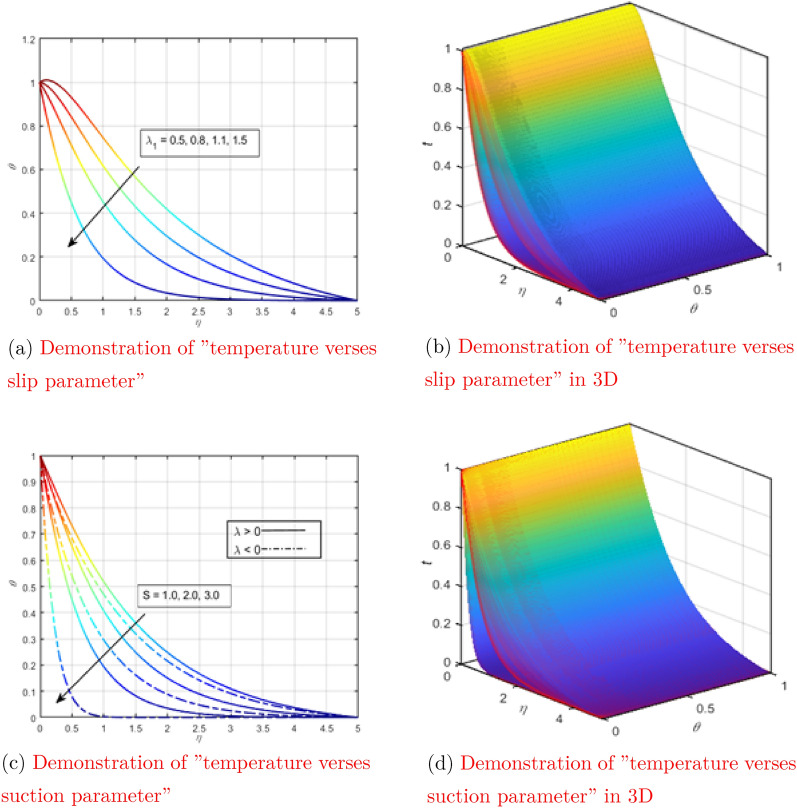
Exhibition of the influence of physical parameters on physical quantities (see Appendix A).

**Figure 6:**
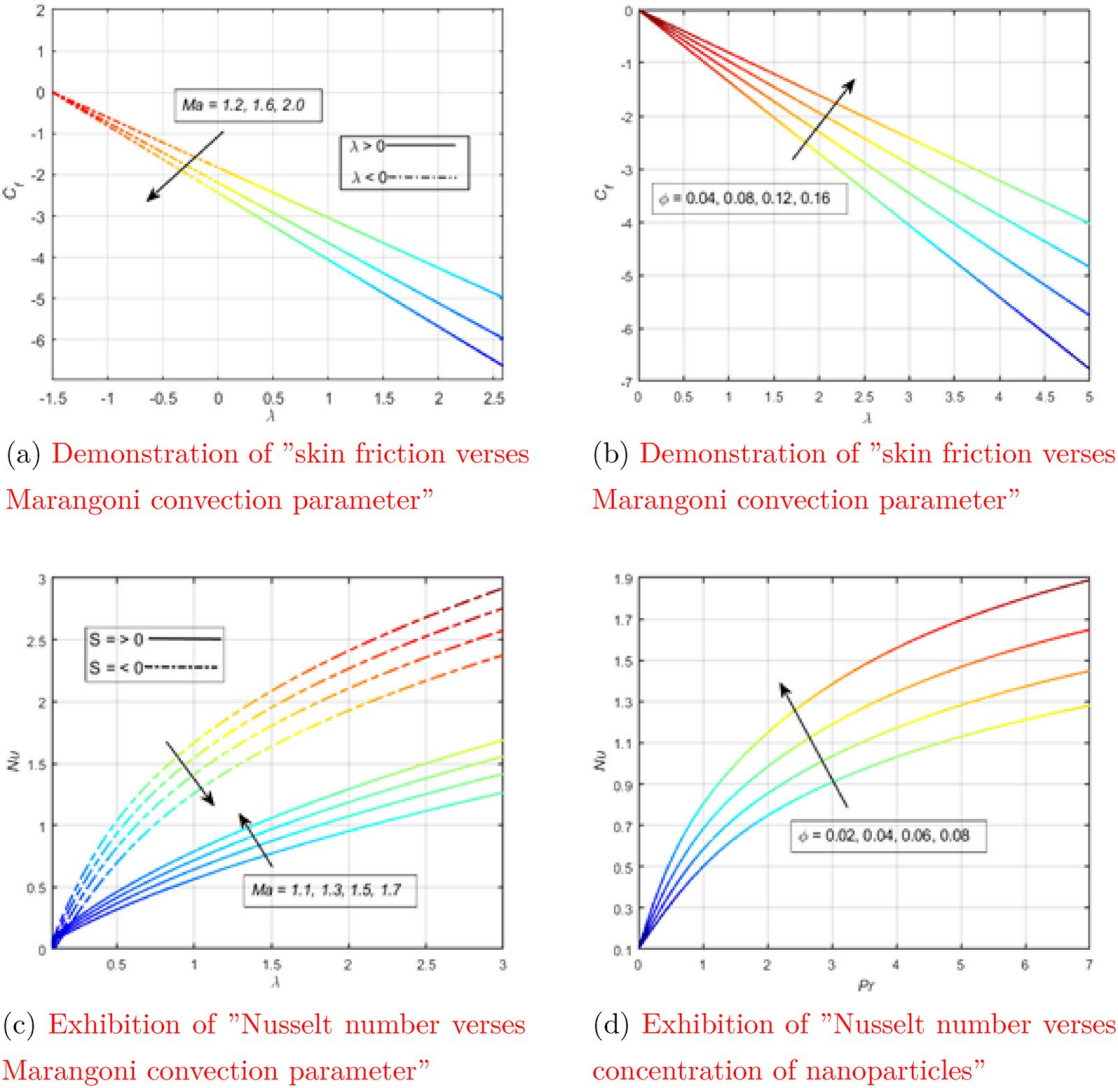
Exhibition of the influence of physical parameters on physical quantities (see Appendix A).

In this part, the influence of the corresponding parameters of the problem being solved on temperature and speed is investigated. To do this, some images are created by changing the value of a parameter in a certain range, and other parameters such as: $A (=\frac{\rho _f \mu _{nf}}{\rho _{nf} \mu _{f}}) = 0.5$, and $B (=\frac{\sigma _{nf} \rho _f}{\sigma _f \rho _{nf}}) = 0.4$, $k_1 (=\frac{k_{nf}}{k_f})= 0.1$, $\tau (=\frac{(\rho c_p)_{nf}}{(\rho c_p)_{f}})= 0.5$, $Pr =6.0674 \; \text {(for water Pr = 6.0674)}$, $\lambda _1 = 0.5$, $S=0.5$, Ma= 0.8, and $\phi = 0.05$ are unchanged. We found that the results are consistent and have significant parallels with previous work (see Table 1), assuming that the flow model is physically stable and the numerical solution method, in particular the “bvp4c” scheme, is correct. Table 2 shows the main thermophysical properties of the base fluid and nanoparticles^40^. Figures 2, 3, 4, 5, 6 and Table 1 are constructed using the bvp4c MATLAB code provided in Appendix A.

**Table 1:** Comparison of the results by calculating $-\theta ^{\prime }(0)$ values for different Prandtl numbers.

Pr	7.0	10	15
Isak et al.^41^	0.8086	1.0000	1.9237
Ganesh et al.^42^	0.808631	1.000000	1.923682
Umar et al.^35^	0.808631	1.000000	1.923682
Current results	0.808630	1.000000	1.923677

**Table 2:** Thermophysical properties of water and copper.

	$C_p \left[ \dfrac{\text{J}}{\text{K} \, \text{kg}}\right]$	$\rho \left[ \dfrac{\text{kg}}{\text{m}^3}\right]$	$k \left[ \dfrac{\text{W}}{\text{K} \; \text{m}}\right]$	$\beta \times 10 ^5 \left[ \dfrac{1}{\text{K}}\right]$
Cu (copper)	385	8933	401	1.67
$\text{H}_2\text{O}$ (pure water)	4179	997.1	0.613	21

Figure 2a shows the behavior of the velocity as a function of $\eta$ when the magnetic parameter is changed in a certain range. The figure above shows that the increase or decrease in velocity occurs in a pattern opposite to the magnetic parameter. The physical reason is that the liquid particles are attracted by the opposite Lorentz force, which causes the liquid particles to move slowly. As a result, the overall fluid velocity decreases. Fig. 2a shows the behavior of the velocity as a function of $\eta$ when the magnetic parameter changes in three-dimensional space. The Fig. 3c shows how the Marangoni convection parameter (Ma) affects fluid velocity without changing all other parameter values. It is noted that the velocity profile changes in the same way as the parameter Ma. The speed increases when this parameter is increased, and slows down when the Marangoni convection parameter (Ma) decreases. The surface tension gradient in liquids is often associated with Marangoni convection. As the Marangoni convection parameter increases, these surface tension gradients develop and liquid particles on the surface rapidly flow into regions of low surface energy. In other words,, the higher the Ma, the lower the viscosity. The smaller the viscous force, the faster the fluid velocity. The Fig. 3d shows the behavior of the velocity as a function of $\eta$ with a change in the Marangoni convection parameter in three-dimensional space.

The behavior of the speed as a function of $\eta$ with a change in the concentration of nanoparticles ($\phi$) in a certain range is shown in Fig. 3e. It should be noted that the velocity values are lower for a concentrated suspension of nanoparticles. A concentrated suspension is more likely to cause collisions between liquid particles. The speed of the particles decreases after colliding with another particle, which causes the speed of the liquid on the graph to decrease. Figure 3f shows the behavior of the velocity as a function of $\eta$ with a change in the concentration of nanoparticles in three-dimensional space. Figure 3a shows a graph of velocity versus suction for two different cases of velocity slip, with all other parameters remaining unchanged. As expected, the velocity was found to be a decreasing function of the suction parameter. Suction, in physical terms, refers to the flow of fluid from a region of low pressure to a region of high pressure. It is quite clear that the liquid decreases as it enters the region of high pressure from the region of low pressure. The Fig. 3b shows the behavior of the velocity as a function of $\eta$ when changing the suction parameter in three-dimensional space.

The curve of the temperature dependence on the magnetic parameter with all other parameters fixed is shown in Fig. 4a. When electromagnetic waves flow through the liquid, the particles vibrate with a higher frequency, increasing the kinetic energy of the particles. Due to the direct relationship between kinetic energy and temperature, the temperature of the liquid rises. Figure 4b shows the behavior of temperature depending on $\eta$ when changing the magnetic parameter in three-dimensional space.

Figure 4c shows the temperature dependence on the Marangoni convection parameter when all other parameters remain constant. The temperature profile is reduced due to the Marangoni convection parameter, as seen in the figure below. Undoubtedly, the speed increases as the viscosity decreases for larger values of Ma. The average kinetic energy of fluid particles is defined as temperature. Therefore, the higher the Ma, the higher the temperature. Figure 4d shows the behavior of temperature as a function of $\eta$ when changing the parameter of Marangoni convection in three-dimensional space. Figure 4e shows the relationship between temperature and nanoparticle concentration, while all other factors remain constant. As you can see in the diagram below, increasing the concentration of nanoparticles improves the temperature profile. This is because when the dispersion of nanoparticles is concentrated, the nanoparticles are more prone to collisions. The temperature of the liquid rises as a result of the huge number of collisions between the nanoparticles. Figure 4f shows the behavior of temperature as a function of $\eta$ when changing the concentration of nanoparticles in three-dimensional space. The dependence of the temperature curve on $\lambda _1$ (thermal relaxation parameter) with all other parameters rigidly set was tested in Fig. 5a. The thermal relaxation parameter tends to decrease the temperature curve, which can be seen in the figure. Figure 5b shows the behavior of temperature as a function of $\eta$ when changing the thermal relaxation parameter in three-dimensional space.

The dependence of the temperature curve on the suction parameter with a rigid setting of all other parameters was tested in Fig. 5c. A decrease in the temperature curve was observed at a high surface suction resolution. Suction has a physical effect on temperature because heat transmission increases as suction on the surface increases. The temperature drops as heat is quickly transmitted from one spot to another. Figure 5d shows the behavior of temperature as a function of $\eta$ when changing the suction parameter in three-dimensional space.

The effect of the Marangoni convection parameter on skin friction by leaving all other parameters unchanged is shown in the Fig. 6a. The Marangoni convection parameter is able to reduce the skin friction coefficient, as can be seen from the attached figure. Figure 6b shows the behavior of the skin friction curve for different values of nanoparticle concentration, keeping the remaining parameters constant. The frictional force between a solid and a liquid is physically referred to as skin friction. In the case of a concentrated suspension of nanoparticles, the interaction of a solid with a liquid is significant and therefore the coefficient of skin friction increases.

The Nusselt number was tested for different values of the Marangoni convection parameter for suction and injection separately in Fig. 6c. The Marangoni convection parameter promotes the Nusselt number during surface suction and lowers the temperature during surface injection. The Nusselt number was tested for various values of the concentration of nanoparticles, the rest of the parameters were set fixed in Fig. 6d. A suspension of nanoparticles has a tremendous ability to transfer heat in the case of a concentrated suspension. The physical reason is that the addition of nanoparticles improves the thermal conductivity of the liquid. Since there is a large amount of coolant, this leads to an increase in the Nusselt number.

## Conclusion

The Cartesian plane has been used to study the Marangoni convection of non-Newtonian MHD nanofluids with temperature-dependent surface tension and Cattaneo–Christov heat flux. Similarity variables were used to transform ruling PDEs to regular ordinary equations. Since bvp4c is a numerical code for a computationally intensive solution, it is used here to track the solution of the developed model. The dependence of some important physical quantities such as speed, skin friction, temperature and Nusselt number on some new physical parameters was checked. Excellent convergence and consistency was found when comparing the results with the previous publication and therefore we can argue that bvp4c is a computational code that is executed with very little effort.

- A direct relationship was found between the fluid velocity and the Marangoni convection parameter, and the inverse relationship was found between the temperature and the Marangoni convection parameter.
- The purpose of the implantation of the magnetic field is to balance the suction rate, which results in a decrease in fluid velocity.
- One of the main goals of this study is to reduce skin friction. Marangoni convection has been found to be one of the inexpensive tools available that can reduce skin friction.
- The advantage of nanoparticle concentration is that it improves the thermal conductivity of the nanofluid, but on the other hand it significantly increases the skin friction. This is a drawback of the high concentration of nanoparticles.
- If there is suction on the surface, the Marangoni convection setting will greatly improve the heat transfer coefficient, but in the case of injection it will tend to lower the heat transfer coefficient.
- The higher the concentration of nanoparticles, the higher the heat transfer rate will be and vice versa.

## Appendix A

function

clc;

% Author: NM Khan

% Physical Parameters

A = 0.5; B = 0.4; $k_1$ = 0.1; tau = 0.5; Pr =6.0674; $lambda_1$ = 0.5; lambda = 0.5; S=0.5; Ma= 0.8, phi= 0.05

% Initial guess

sol = bvpinit(linspace(0,5,100), [0 0 0 0 0]);

% solution in structure form

sol1 = bvp4c( bvpexam2, bcexam2, sol);

lastwarn(‘ new_msgstr ‘);

% x values

x1 = sol1.x;

% s values in row form (s, $s^{\prime }, s^{\prime \prime }$, theta, $theta^{\prime }$)

s1 = sol1.s;

% plot any one of the solutions s1, here we plot theta figure (1)

plot(x1, s1(2, :), ‘ b ‘, ‘ linewidth ‘, 1, ‘ Linesmoothing‘, ‘ on‘);

xlabel(‘ \eta ‘)

ylabel(‘ \f ⌃{\prime} (\eta) ‘)

% grid on

hold all

value = deval(sol1, 0);

% get results upto six digits

vpa(value, 6)

hold on

% Here I define residual of boundary conditions

function res = bcexam2(s0, sinf)

$$res = [s0(1)-S; s0(2) + 2 \star lambda \star Ma \star (1 - phi )$$ ⌃$$(-2.5); s0(4) - 1; sinf(2); sinf(4)];$$

end

% First order ODEs are define here

function ysol = bvpexam2(x,s)

$$ss1 = (1/A) (- s(1)\star s(3) + s(2)$$ ⌃$$2 + B \star M \star s(2));$$

$$ss2 = - (1/(k_1 - tau \star Pr \star s(1)$$ ⌃$$2)) \star tau \star Pr \star (s(1) \star s(5) - 2 \star s(2) \star s(4) + lambda_1 \star (5 \star s(1) \star s(2) \star s(5) + 2 \star s(1) \star s(3) \star s(4) - s(2)$$ ⌃$$2 \star s(4)));$$

ysol = [s(2); s(3); ss1; s(5); ss2];

end

end
